# Supplementary material for: Modeling glioblastoma heterogeneity as a dynamic network of cell states
Source: Mol Syst Biol. 2021 Sep 16;17(9):e10105. doi: 10.15252/msb.202010105 (PMC8444284; doi:10.15252/msb.202010105)
Supplement: Supplementary file 6 — Source Data for Figure 5 [file MSB-17-e10105-s004.zip › Figure5A_sourcedata/GSEA_3017/hallmarks_stateF.GseaPreranked.1621934628066/HALLMARK_HEME_METABOLISM.html]

Details for gene set HALLMARK\_HEME\_METABOLISM[GSEA]

|  || Dataset | state33017 |
| Phenotype | NoPhenotypeAvailable |
| Upregulated in class | na\_pos |
| GeneSet | HALLMARK\_HEME\_METABOLISM |
| Enrichment Score (ES) | 0.3657851 |
| Normalized Enrichment Score (NES) | 1.6976466 |
| Nominal p-value | 0.027829314 |
| FDR q-value | 0.03294852 |
| FWER p-Value | 0.33 |
Table: GSEA Results Summary

  

Fig 1: Enrichment plot: HALLMARK\_HEME\_METABOLISM      
 Profile of the Running ES Score & Positions of GeneSet Members on the Rank Ordered List

  

| PROBE | GENE SYMBOL | GENE\_TITLE | RANK IN GENE LIST | RANK METRIC SCORE | RUNNING ES | CORE ENRICHMENT || 1 | SDCBP |  |  | 27 | 1.054 | 0.0592 | Yes |
| 2 | BTG2 |  |  | 124 | 0.708 | 0.0639 | Yes |
| 3 | DAAM1 |  |  | 175 | 0.618 | 0.0832 | Yes |
| 4 | KAT2B |  |  | 193 | 0.594 | 0.1158 | Yes |
| 5 | BLVRB |  |  | 235 | 0.565 | 0.1356 | Yes |
| 6 | CA2 |  |  | 243 | 0.558 | 0.1703 | Yes |
| 7 | ENDOD1 |  |  | 262 | 0.543 | 0.1990 | Yes |
| 8 | TFRC |  |  | 270 | 0.537 | 0.2322 | Yes |
| 9 | LAMP2 |  |  | 308 | 0.506 | 0.2498 | Yes |
| 10 | NUDT4 |  |  | 318 | 0.502 | 0.2797 | Yes |
| 11 | SEC14L1 |  |  | 465 | 0.445 | 0.2441 | Yes |
| 12 | LPIN2 |  |  | 488 | 0.438 | 0.2639 | Yes |
| 13 | LRP10 |  |  | 492 | 0.438 | 0.2922 | Yes |
| 14 | LMO2 |  |  | 497 | 0.436 | 0.3199 | Yes |
| 15 | TMEM9B |  |  | 539 | 0.417 | 0.3297 | Yes |
| 16 | BNIP3L |  |  | 565 | 0.409 | 0.3461 | Yes |
| 17 | YPEL5 |  |  | 583 | 0.403 | 0.3658 | Yes |
| 18 | ALDH1L1 |  |  | 744 | 0.361 | 0.3181 | No |
| 19 | FOXO3 |  |  | 801 | 0.346 | 0.3163 | No |
| 20 | CLCN3 |  |  | 937 | 0.320 | 0.2772 | No |
| 21 | TNRC6B |  |  | 965 | 0.316 | 0.2864 | No |
| 22 | ALDH6A1 |  |  | 987 | 0.311 | 0.2981 | No |
| 23 | BCAM |  |  | 1049 | 0.300 | 0.2909 | No |
| 24 | ATP6V0A1 |  |  | 1082 | 0.295 | 0.2965 | No |
| 25 | SLC7A11 |  |  | 1131 | 0.288 | 0.2943 | No |
| 26 | IGSF3 |  |  | 1246 | 0.275 | 0.2616 | No |
| 27 | SLC6A9 |  |  | 1278 | 0.271 | 0.2659 | No |
| 28 | SMOX |  |  | 1305 | 0.268 | 0.2723 | No |
| 29 | ARHGEF12 |  |  | 1379 | 0.259 | 0.2570 | No |
| 30 | RNF19A |  |  | 1429 | 0.255 | 0.2521 | No |
| 31 | VEZF1 |  |  | 1732 | -0.331 | 0.1385 | No |
| 32 | TOP1 |  |  | 1749 | -0.340 | 0.1543 | No |
| 33 | GLRX5 |  |  | 1778 | -0.351 | 0.1655 | No |
| 34 | HDGF |  |  | 2124 | -0.709 | 0.0581 | No |
Table: GSEA details [plain text format]

  

Fig 2: HALLMARK\_HEME\_METABOLISM: Random ES distribution      
 Gene set null distribution of ES for **HALLMARK\_HEME\_METABOLISM**
